# Serum xanthophyll carotenoids are associated with estimated glomerular filtration rate in an aged cohort

**DOI:** 10.1038/s41598-019-53674-5

**Published:** 2019-11-19

**Authors:** Declan Browne, Michael A. Williams, Alexander P. Maxwell, Bernadette McGuinness, Peter Passmore, Giuliana Silvestri, Jayne V. Woodside, Gareth J. McKay

**Affiliations:** 1 Centre for Public Health, Queen’s University Belfast, Belfast, UK; 2 Centre for Medical Education, Queen’s University of Belfast, Belfast, UK; 3 Centre for Experimental Medicine, Queen’s University of Belfast, Belfast, UK

**Keywords:** Nephrology, Kidney

## Abstract

Progressive renal decline is associated with increasing oxidative stress. However, the majority of studies have investigated endogenous antioxidants in predominantly advanced stages of kidney disease. Many traditional risk factors associated with renal dysfunction have been linked with cognitive decline as the kidneys and brain share comparable anatomic and haemodynamic characteristics that leave them susceptible to common pathogenic mechanisms. The objective of this study was to examine serum dietary antioxidants and their association with renal function characterised by estimated glomerular filtration rate (eGFR) in a cross-sectional analysis of 570 participants. High performance liquid chromatography quantified serum levels of retinol, α-tocopherol, γ-tocopherol and six carotenoids (α-carotene, β-carotene, β-cryptoxanthin, lutein, lycopene and zeaxanthin) in participants. Multiple regression analyses were used to evaluate associations while adjusting for potential confounders. A sensitivity analysis was performed in cognitively-intact participants only. Serum levels of the xanthophyll carotenoid lutein were positively associated with eGFR in analyses adjusted for age (years), gender, smoking, *APOE4* status and Alzheimer’s disease. Retinol was inversely associated with eGFR, although was no longer significant in the smaller sensitivity analysis. Our findings identify significant associations between the xanthophyll carotenoids and eGFR. Further investigations are required to confirm these findings.

## Introduction

Renal dysfunction remains a significant public health concern given its potential to progress to chronic kidney disease (CKD) and end-stage renal disease (ESRD) without intervention. CKD is estimated to affect 11–13% of the population and is forecast to become the fifth leading cause of death globally by 2040^1^. Progressive renal dysfunction is identified typically through repeated measures of serum creatinine and estimated glomerular filtration rate (eGFR). CKD is categorised into five stages based on eGFR range. CKD stages 3 or higher are defined by eGFR measurements below 60 mL/min/1.73 m² for at least 3 months^2^. Interventions that improve or conserve renal function are clinically valuable, as they may delay or prevent CKD progression to ESRD and the requirement for costly treatments such as dialysis and/or kidney transplantation^3^. CKD is also associated with higher risk of cardiovascular events, hospitalisation and premature death^4^.

Many of the traditional risk factors associated with renal dysfunction have also been linked with cognitive decline and Alzheimer’s disease (AD) and the prevalence of mild cognitive impairment (MCI) has been estimated to be twice as high in those with CKD compared to the general population, with estimates ranging between 30–63%^5,6^. As end-organs, the kidneys and brain have comparable anatomic and haemodynamic characteristics which leave them susceptible to common pathogenic mechanisms^7^. As such, CKD and AD share similar risk factor profiles comprising hypertension, diabetes mellitus and hypercholesterolaemia, with similar prevalence of small-vessel disease^8^. Furthermore, variation within apoE, a protein crucial to lipoprotein metabolism, including reverse cholesterol transport, hepatic clearance of chylomicrons and very low-density lipoprotein remnants, has been reported in association with chronic kidney disease progression, independent of other risk factors^9^. In addition, apoE4 has been consistently shown to exhibit poorer antioxidant properties in comparison to apoE3 and apoE2^10^. Multiple pathways may contribute to the close association between renal dysfunction and cognitive impairment including shared mechanisms resulting in vascular pathophysiology^11^. Depression, neurological sequelae and cognitive impairment are also severe co-morbidities of CKD that significantly reduce quality of life^12^.

In recent years, non-traditional risk factors such as inflammation and oxidative stress (OS) have been implicated in the pathogenesis of both CKD and cognitive decline^13^. While the precise mechanisms remain unclear, there is substantial evidence that AD is associated with OS and altered serum dietary antioxidant status^14,15^. Similarly, OS has been implicated as a pathogenic mechanism in CKD that may directly induce damage to the kidney’s microstructures or indirectly through inflammation and endothelial dysfunction^16^. Whether these changes are secondary to diminished antioxidant intake or the excessive generation of reactive oxygen species remains uncertain. However, an association between OS and eGFR has been established and the detrimental consequences of OS in CKD have been associated with increased mortality in patients^17,18^. Importantly, malnourished individuals with poor renal function exhibit increased evidence of OS compared to those receiving adequate nutrition, highlighting the importance of dietary antioxidants in those with poor renal function^19^. However, the majority of studies investigating OS and antioxidant defences in renal disease have focused largely on ESRD and endogenous markers of OS^20^.

The aim of this study was to investigate the association between serum antioxidant status and renal function, as measured by eGFR, while controlling for AD status. Given advanced CKD (stages 4 and particularly stage 5) could impact on dietary antioxidant levels secondary to reduced dietary intake or increased losses (from dialysis therapies), we sought to recruit older adults who were known not to be suffering from advanced chronic kidney disease and were not attending nephrology clinics (to avoid confounding from late CKD stages). We examined serum concentrations of nine dietary antioxidants including three antioxidant vitamins (retinol, α-tocopherol, γ-tocopherol) and six carotenoids (α-carotene, β-carotene, β-cryptoxanthin, lutein, lycopene and zeaxanthin).

## Methods

### Study population

A cross-sectional study of 570 participants was conducted to assess the association between eGFR and serum antioxidant status. Recruitment and testing of all participants was undertaken between August 2006 and 2008 by one investigator (M.A.W.) and has been described elsewhere^21^. The population consisted of prevalent AD cases and unmatched cognitively intact individuals. Participants with AD were identified and recruited from memory clinics in Belfast City Hospital, UK and Knockbracken Healthcare Park, UK, or from records of previous attendance at the same clinics. Diagnosis of AD was made by a senior consultant using the National Institute of Neurological and Communicative Disorders and Stroke and the Alzheimer’s Disease and Related Disorders Association (NINCDS ADRDA) criteria^22^. Individuals were excluded if they had a diagnosis of dementia not attributable to AD. Various recruitment strategies were used to identify cognitively intact participants. These strategies included asking carers of patients attending any outpatient clinic in the study hospital to participate. A university press-release resulted in the recruitment of further volunteers. Participants considered to be cognitively intact were excluded if they were aged under 65 years, had a history of any neurological disease or a Mini-Mental State examination (MMSE) score below 26/30.

All participants provided written informed consent prior to study entry, which adhered to the principles of the Declaration of Helsinki. Clinical governance and ethical approval was obtained prior to study commencement and the research was undertaken in accordance with relevant guidelines under ethical approval by the Office for Research Ethics Committees Northern Ireland.

### Data collection

Upon enrolment, all participants underwent an examination that included blood pressure measurement and provision of a blood sample. Standard venepuncture was used to draw blood and determine serum creatinine for eGFR using the CKD-EPI equation^23^.

Serum levels of nine dietary antioxidants (retinol, α-tocopherol, γ-tocopherol, α-carotene, β-carotene, β-cryptoxanthin, lutein, lycopene and zeaxanthin) were determined by high performance liquid chromatography (HPLC) with diode array detection^24^. Samples were coded and stored at −80 °C following collection and were batch-analysed in a blinded manner. Internal quality control samples were incorporated into every run and validation was conducted against National Institute of Standards and Technology standard reference material.

Participants were further assessed through the completion of questionnaires via interviews with themselves or their carer when appropriate. Use of medication and co-morbidities were noted as present or absent following determination by self-report or consultation of medical notes. Smoking history was calculated as a cumulative dose in pack years.

### Statistical analysis

All statistical analyses were conducted using SPSS statistics version 23 (IBM Corp. Armonk, NY). Summary statistics were presented as mean (standard deviation) for continuous data, all of which was normally distributed, and relative frequencies (percentage) by group, for categorical variables. These were presented for all participants and separately for the cognitively intact sub-group.

Linear regression models, with eGFR as the dependent variable and serum concentrations of the antioxidant vitamins and carotenoids entered individually as continuous independent variables, were used to calculate regression coefficients with 95% confidence intervals (CI). The variation in eGFR associated with a unit increase in concentration of each of the nine dietary antioxidants examined was determined. These coefficients were calculated before and after adjustment for confounders.

Variables were included in the multiple regression analysis if a significant association (*P* < 0.05) was demonstrated on univariate analysis or if there was an established biological association with eGFR. The final model was determined using a backward selection procedure that retained only variables which showed a significant association with eGFR. Thus, the final model used to calculate the regression coefficients contained age (years), gender and smoking status. AD was also retained due to the composition of the study population.

A sensitivity analysis was performed to investigate any potential confounding effects of AD status in the relationship between eGFR and serum antioxidant status. In a sensitivity analysis, multiple linear regression was performed in cognitively intact individuals only, with eGFR as the dependent variable and serum concentrations of the dietary antioxidants individually entered as independent variables along with age (years), gender and smoking status.

## Results

### Study population

The summary clinical and demographic characteristics of the study population (*n* = 570) and the cognitively intact subgroup (*n* = 317) are presented in Table 1. The mean age and standard deviation (SD) of the study population was 78.1 (7.4) years and comprised 216 males (38%). The mean (SD) eGFR across the population was 37.4 mL/min/1.73 m² (8.9) with a mean (SD) systolic blood pressure of 139 (18) mm Hg. Two-hundred and fifty-three participants (44%) had a diagnosis of AD. Three-hundred and twenty-one participants (56%) reported never having smoked. Fifty-nine participants (11%) had diabetes mellitus and 221 (41%) had a diagnosis of hypertension. One-hundred participants (19%) reported regularly taking thiazide diuretics with a further 33 (6.2%) taking regular non-steroidal anti-inflammatory drugs.

**Table 1:** Summary statistics of subject characteristics.

Characteristic	All (n = 570)	Cognitively normal sub-group (n = 317)
Mean age, years (SD)	78.1 (7.4)	76.5 (6.7)
Male, n (%)	216 (38)	125 (39)
Mean systolic blood pressure, mmHg (SD)	139 (18)	144 (18)
Mean eGFR CKD-EPI, ml/min/1.73 m² (SD)	37.4 (8.9)	37.3 (8.4)
Never smoked, n (%)	321 (56)	191 (60)
Mean MMSE (SD)	24.4 (6.8)	28.8 (1.2)
Presence of at least 1 *APOE4* allele, n (%)	240 (45)	71 (25)
Education – left school at 14, n (%)	274 (51)	138 (45)
Diabetes mellitus, n (%)	59 (11)	37 (12)
Hypertension, n (%)	221 (41)	131 (43)
Cardiovascular disease, n (%)	129 (23)	76 (25)
Cerebrovascular disease, n (%)	70 (12)	38 (12)
Hypercholesterolaemia, n (%)	216 (40)	124 (41)
Aspirin and/or clopidogrel, n (%)*	229 (43)	116 (39)
Antacids, n (%)*	144 (27)	71 (24)
Thiazide, n (%)*	100 (19)	54 (19)
Non-thiazide diuretics, n (%)*	57 (11)	24 (8.2)
NSAIDs, n (%)*	33 (6.2)	22 (7.3)
Thyroxine, n (%)*	59 (11)	30 (10)
CCBs, n (%)*	62 (12)	28 (9.7)
Beta-blockers, n (%)*	118 (23)	75 (26)

Abbreviations: SD, standard deviation; MMSE, Mini-Mental State Examination; eGFR CKD-EPI, estimated glomerular filtration rate calculated using the CKD-EPI equation; NSAIDS, non-steroidal anti-inflammatory drugs; CCBs, calcium channel blockers. *Medications recorded with a frequency >5%.

### Association between eGFR and serum antioxidant levels

The association of serum levels of the nine dietary antioxidants with eGFR for the study population (*n* = 570) are presented in Table 2. In univariate analyses, retinol (β = −1.363, 95% CI: −2.595 to −0.131, *P* = 0.03) showed a significant inverse association with eGFR. In contrast, lutein (β = 0.050, 95% CI: 0.023 to 0.077, *P* = < 0.001) and zeaxanthin (β = 0.123, 95% CI: 0.012 to 0.235, *P* = 0.03) demonstrated significant positive associations with eGFR. Both vitamin E isoforms and the remaining carotenoids demonstrated no significant association with eGFR in univariate analyses.

**Table 2:** Association of serum antioxidants with eGFR in all study participants.

Antioxidant	Unadjusted analysis			Adjusted analysis^†^		
	β*	95% confidence intervals	*P*	β*	95% confidence intervals	*P*
Retinol (μmmol/L)	−1.363	−2.595, −0.131	0.03	−1.436	−2.598, −0.274	0.02
γ-Tocopherol (μmmol/L)	−0.069	−0.437, −0.299	0.71	−0.035	−0.398, 0.329	0.85
α-Tocopherol (μmmol/L)	0.013	−0.078, 0.103	0.79	0.048	−0.041, 0.136	0.29
Lutein (mmol/L)	0.050	0.023, 0.077	<0.01	0.041	0.015, 0.068	<0.01
Zeaxanthin (mmol/L)	0.123	0.012, 0.235	0.03	0.097	−0.014, 0.207	0.09
β−Cryptoxanthin (mmol/L)	0.030	−0.029, 0.089	0.32	0.048	−0.012, 0.107	0.12
α-Carotene (mmol/L)	0.019	−0.026, 0.065	0.41	0.028	−0.020, 0.075	0.25
β-Carotene (mmol/L)	0.005	−0.003, 0.014	0.21	0.008	0.000, 0.017	0.05
Lycopene (μmmol/L)	0.048	−1.622, 1.718	0.96	0.145	−1.637, 1.927	0.87

*Average increase in each serum antioxidant level per unit increase in eGFR.

^†^Multiple regression analysis adjusted for age (years), gender, smoking (pack-years), the number of *APOE4* alleles and Alzheimer’s disease status.

In the analyses of all participants adjusted for age (years), gender, smoking, number of *APOE4* alleles and AD status, retinol (β = −1.436, 95% CI: −2.598 to −0.274, *P* = 0.02) and lutein (β = 0.041, 95% CI: 0.015 to 0.068, *P* = < 0.001) remained significantly associated with eGFR. The remaining antioxidants were not significant in the multivariate analyses. Associations between antioxidant measures and eGFR were also considered separately for those individuals with no copies of the *APOE4* allele (n = 292) and 1 or 2 copies of the *APOE4* allele (n = 240). Although the direction of effect was maintained, no significant associations were detected in participants with no copies of *APOE4* (Table 3). In individuals with one or two copies of *APOE4*, lutein (β = 0.059, 95% CI: 0.016 to 0.101, P < 0.01), zeaxanthin (β = 0.253, 95% CI: 0.040 to 0.466, P = 0.02) and β-carotene (β = 0.023, 95% CI: 0.006 to 0.039, P < 0.01) were significantly associated with eGFR (Table 4).

**Table 3:** Association of serum antioxidant with eGFR in individuals with no copies of the *APOE4* allele.

Antioxidant	Unadjusted analysis			Adjusted analysis^†^		
	β*	95% confidence intervals	*P*	β*	95% confidence intervals	*P*
Retinol (μmmol/L)	−1.270	−2.639, 0.099	0.07	−1.235	−2.521, 0.051	0.06
γ-Tocopherol (μmmol/L)	−0.024	−0.585, 0.536	0.93	0.036	−0.503, 0.575	0.89
α-Tocopherol (μmmol/L)	−0.023	−0.124, 0.078	0.65	0.005	−0.092, 0.102	0.92
Lutein (mmol/L)	0.038	0.002, 0.074	0.04	0.029	−0.005, 0.063	0.10
Zeaxanthin (mmol/L)	0.058	−0.079, 0.194	0.41	0.027	−0.103, 0.158	0.68
β-Cryptoxanthin (mmol/L)	0.018	−0.052, 0.088	0.62	0.030	−0.038, 0.097	0.39
α-Carotene (mmol/L)	−0.008	−0.065, 0.050	0.79	0.005	−0.050, 0.061	0.85
β-Carotene (mmol/L)	0.000	−0.011, 0.010	0.99	0.002	−0.008, 0.012	0.65
Lycopene (μmmol/L)	−1.028	−3.104, 1.047	0.33	−0.634	−2.631, 1.363	0.53

*Average increase in each serum antioxidant level per unit increase in eGFR.

^†^Multiple regression analysis adjusted for age (years), gender, smoking (pack-years) and Alzheimer’s disease status.

**Table 4:** Association of serum antioxidant with eGFR in individuals with one or two copies of the *APOE4* allele.

Antioxidant	Unadjusted analysis			Adjusted analysis^†^		
	β*	95% confidence intervals	*P*	β*	95% confidence intervals	*P*
Retinol (μmmol/L)	−2.001	−4.845, 0.842	0.17	−2.409	−5.111, 0.294	0.08
γ-Tocopherol (μmmol/L)	−0.185	−0.718, 0.348	0.50	−0.095	−0.596, 0.407	0.71
α-Tocopherol (μmmol/L)	0.160	−0.050, 0.370	0.14	0.225	0.001, 0.450	0.05
Lutein (mmol/L)	0.062	0.018, 0.106	0.006	0.059	0.016, 0.101	<0.01
Zeaxanthin (mmol/L)	0.233	0.028, 0.437	0.03	0.253	0.040, 0.466	0.02
β-Cryptoxanthin (mmol/L)	0.043	−0.080, 0.166	0.49	0.097	−0.030, 0.224	0.13
α-Carotene (mmol/L)	0.090	−0.007, 0.187	0.07	0.083	−0.012, 0.178	0.09
β-Carotene (mmol/L)	0.017	0.000, 0.033	0.05	0.023	0.006, 0.039	<0.01
Lycopene (μmmol/L)	3.741	−0.352, 7.834	0.07	2.975	−1.020, 6.970	0.14

*Average increase in each serum antioxidant level per unit increase in eGFR.

^†^Multiple regression analysis adjusted for age (years), gender, smoking (pack-years) and Alzheimer’s disease status.

### Sensitivity analysis

The sensitivity analysis was performed in cognitively intact participants only (Table 5). This analysis was similarly adjusted for age (years), gender, number of *APOE4* alleles and smoking status. Lutein (β = 0.035, 95% CI: 0.004 to 0.066, *P* = 0.03) remained significantly associated with eGFR. Retinol (β = −1.152, 95% CI: −2.422 to 0.118, *P* = 0.08) was no longer significantly associated with eGFR in this smaller subset of individuals. There were no significant associations between eGFR and vitamin E or the remaining carotenoids.

**Table 5:** Sensitivity association analysis of eGFR with serum antioxidant in cognitively intact participants only.

Antioxidant	β*†	95% confidence intervals	*P*
Retinol (μmmol/L)	−1.152	−2.422, 0.118	0.08
γ-Tocopherol (μmmol/L)	0.198	−0.370, 0.765	0.50
α-Tocopherol (μmmol/L)	−0.005	−0.097, 0.086	0.91
Lutein (mmol/L)	0.035	0.004, 0.066	0.03
Zeaxanthin (mmol/L)	0.067	−0.055, 0.188	0.28
β-Cryptoxanthin (mmol/L)	0.039	−0.027, 0.105	0.25
α-Carotene (mmol/L)	0.002	−0.049, 0.052	0.95
β-Carotene (mmol/L)	0.002	−0.008, 0.011	0.74
Lycopene (μmmol/L)	−0.234	−2.046, 1.577	0.80

*Average increase in each serum antioxidant level per unit increase in eGFR.

^†^Multiple regression analysis adjusted for age (years), sex and smoking (pack-years) and the number of *APOE4* alleles.

## Discussion

This large cross-sectional analysis examined the relationship between serum levels of nine dietary antioxidants and renal function recording significant associations between the xanthophyll serum carotenoid lutein and eGFR. A significant negative association was also detected between eGFR and serum retinol although this was not significant in the sensitivity analysis conducted on cognitively intact participants only. Our results support the findings reported from interventional trials of exogenous antioxidants in renal dysfunction with no association detected between vitamin E and renal function^25^. Given our observations, the potential interventional benefits of the significantly associated serum xanthophyll carotenoids with renal function, especially in those carrying the *APOE4* allele, warrants further investigation.

Several potential limitations of our study should be considered. Firstly, study participants were recruited as part of a case-control study initially comparing AD to cognitively intact participants^26^. However, the potential confounding of AD status was minimised through adjustment in our analyses in addition to the separate sensitivity analysis undertaken that included cognitively intact participants only. No independent association between eGFR and AD status was detected in this study population^27^. Despite observations of similar effect sizes in both the sensitivity analysis of cognitively intact individuals and the full analysis, the loss of a significant association between eGFR and retinol in the sensitivity analysis only, may result from a smaller sample size given the reduced power to detect association.

Secondly, convenience sampling methods were used to recruit participants. While this enabled recruitment of a large sample size, it may have led to sampling bias in participant selection. It may also partially explain the lower mean and narrower range of eGFR measurements than would normally be expected in a similar population-based study of elderly participants^28^. Future studies should ideally investigate a broader population with a higher mean and wider range of eGFR values.

Thirdly, the use of a single measure of eGFR derived from a serum creatinine-based equation may limit the strength of assumptions made from the data. Ideally, serial measurements should be used and other studies have utilised cystatin-C which may offer a more precise estimate of renal function in an elderly population with a high prevalence of co-morbidities^2^.

Finally, it is possible that other factors that influence eGFR or antioxidant status were not sufficiently considered within our data. A potentially important omission may be information on participant dietary intake and supplement use, which could have been recorded through food diaries or other questionnaire-based methods. However, the value of these methods to examine the relationship between diet and disease risk may have been limited by the potential recall bias of some of the study participants, should it have been used.

The cross-sectional nature of our study precludes assumptions about the temporal relationship of any associations observed between eGFR and serum antioxidant status. Therefore, dietary information may have been useful in determining whether altered levels of serum antioxidants are truly a result of declining renal function or reduced dietary consumption. This would be particularly important in the context of CKD where an estimated 50% of patients have restricted diets that influence circulating antioxidant levels^29^. Our investigation of a non-CKD population is likely to have removed the confounding effects of any dietary restrictions. While an established association between AD and poor dietary intake has been demonstrated previously, the sensitivity analysis undertaken is also likely to have mitigated its effects^30^. As such, the strength of our conclusions may be limited without a valid measure of dietary intake and supplement use. Furthermore, Viggiano and colleagues recently suggested individuals with MCI and CKD may represent a distinct or extreme phenotype in comparison to those with MCI and no renal impairment in the general population^31^. The association between MCI and CKD is most evident in individuals with late CKD (stages 4 and 5) and in persons receiving chronic dialysis treatment^31^.

Our study also had several strengths including the large sample size and the well characterised study population enabling appropriate adjustment for confounding effects. Importantly, the use of HPLC to determine serum levels of antioxidants is an objective measurement that provides a reliable evaluation of the relationship between dietary constituents and disease markers, including eGFR^32^. Separate analyses were also undertaken to evaluate associations between eGFR and antioxidant measures according to APOE status which identified additional associations between eGFR and zeaxanthin and β-carotene in individuals with at least one *APOE4* allele. The apoE4 variant has consistently shown poorer antioxidant properties in comparison to apoE3 and apoE2^10^. Geographic variation in *APOE* allele frequencies and all-cause mortality has been reported previously and may account for some of the variation in the associations identified between studies^33^. Improved understanding of the genetic architecture that underpins antioxidant bioavailability and bioactivity will enable personalised and more effective recommendations for antioxidant intake through dietary intervention or nutritional supplementation^34^.

Multiple abnormalities of redox status have been reported in patients with progressive renal dysfunction^35^. Many of these studies have shown a gradual elevation of oxidative damage and a reduction in endogenous antioxidant defences as disease progresses^20^. However, despite the biological plausibility of potential benefit, other dietary antioxidants have been inadequately investigated with regard to renal dysfunction and studies have been limited by low sample size and inconsistent findings^36^.

Retinol is one of several compounds which comprise vitamin A and is physiologically held within strict parameters due to its important role in cell metabolism^37^. It is commonly found in food sources such as liver, fish and eggs. Our findings demonstrate a significant inverse association between serum levels of retinol and eGFR which is consistent with previous findings^38,39^. Furthermore, patients with high retinol levels have been shown to have a greater risk of developing CKD compared to those with lower levels^40^. Our findings support evidence that raised serum retinol levels may represent a biomarker of CKD risk. However, the temporal relationship of this association remains uncertain and the reduced significance of retinol in our sensitivity analysis of cognitively intact individuals only raises the potential confounding influence of AD status on the association observed.

Carotenoids are lipophilic phytochemicals which can be divided into two sub-classes according to their polarity: non-polar carotenes (including α-carotene, β-carotene and lycopene) and polar xanthophylls (including lutein, zeaxanthin and β-cryptoxanthin)^41^. Each carotenoid exhibits individual antioxidant properties depending on its unique functional group and magnitude of conjugated double-bonds. Our findings support the limited evidence available for the carotene subclass insofar as α-carotene and β-carotene show no significant association with eGFR^39,42^. This may be due to their status as pro-vitamin A molecules that are guided by a negative feedback from high retinol levels in renal disease reducing their conversion to retinol^43^. Our findings may indicate that the relationship of α-carotene and β-carotene to retinol balances their oxidant-scavenging effects and produces unaltered levels overall in reduced kidney function.

Interestingly, our study also found no significant association between serum levels of lycopene and eGFR. Lycopene has an unsaturated chemical structure that provides a powerful ability to quench free radicals *in vitro* and is the predominant carotenoid in human serum^44^. Low circulating lycopene levels have furthermore been linked with ESRD, although these findings may have been influenced by the complicated effects of haemodialysis^39^. The non-significance of lycopene in our adjusted analysis (*P* = 0.87) may reflect what Ghezzi *et al*. describe as a common discrepancy between *in vitro* antioxidant studies and findings in human subjects, highlighting the necessity for improved understanding of the mechanisms that influence antioxidant defences and renal function^45^.

Evaluation of the xanthophyll subclass and renal function has been limited. One small study reported unaltered plasma lutein levels in a small number of patients (n = 66) with CKD^38^. Xanthophylls are commonly found in dark green leafy vegetables and account for less than 40% of the total carotenoid concentration in human plasma^46^. The lutein isomer investigated in this study was significantly associated with eGFR in all our analyses and highlights the importance of the need for further research into the role of xanthophylls and their influence on renal function, which is poorly understood.

Vitamin E is a principal dietary antioxidant with powerful *in vitro* free radical quenching properties. The term vitamin E refers to a group of eight isoforms consisting of four tocopherols and four tocotrienols which are prevalent in vegetable oils, nuts and seeds in varying proportions^47^. Our study found no significant association between eGFR and either α-tocopherol or γ-tocopherol, supporting the findings of several observational studies that examined plasma levels of vitamin E (predominantly α-tocopherol) in pre-dialysis CKD patients^39,48^. Similar findings have been reported in haemodialysis patients when blood samples were taken prior to the commencement of treatment^49^. In contrast, other studies have reported reduced levels of vitamin E in CKD patients when compared to healthy controls^50^.

Nevertheless, the potential clinical benefit of antioxidant therapy in improving renal function in pre-dialysis CKD patients following the administration of intravenous antioxidants including iron has been reported^36^. A recent systematic review also indicated that antioxidant agents significantly decreased albuminuria in diabetic kidney disease (DKD) patients, despite significant limitations of small study size and heterogeneity^51^. The limited number of interventional studies of dietary antioxidants in CKD have been largely focused on vitamin E isoforms (mainly alpha-tocopherol) and late stages of disease with little success. Ramos *et al*. found no changes in OS biomarkers in pre-dialysis CKD patients receiving mixed tocopherols and lipoic acid^25^. In haemodialysis patients, exogenous antioxidant trials have had similarly poor outcomes^52^. The failure of these trials may be particularly important, as haemodialysis has been shown to exacerbate oxidative stress and the diminution of antioxidant defences although the benefits of tocopherol supplementation remains unproven^20^. Interestingly, a recent clinical trial reported the beneficial effects of supplementation with the poorly-studied tocotrienol-rich vitamin E from palm oil (Tocovid) in DKD patients. Tocotrienols have been shown to have significantly more potent antioxidant properties compared to tocopherol with superior antiglycaemic, anticholesterolaemic, anti-inflammatory, neuroprotective, and cardioprotective properties^53^.

The failure of intervention trials contrasts with studies examining the influence of general dietary trends on renal function and emerging prospective evidence. A recent systematic review into the effects of dietary interventions in CKD found evidence that some general dietary trends were associated with higher eGFR and reduced risk of ESRD and its associated risk factors^54^. Despite uncertainty around the precise mechanisms and nutrients associated with improved renal function and reduced mortality, increased circulating antioxidant levels have been proposed as a probable explanation^55^.

Similarly, evidence from prospective studies suggest dietary carotenoids may provide a beneficial role, either as part of a multi-faceted lifestyle or independently, given albuminuria has been associated with diminished levels of carotenoid-intake^56^. A recently published study by Hirahatake *et al*. investigated the relationship between dietary intake and renal function over five years^57^. Their study found that higher serum carotenoid levels were inversely associated with decline in kidney function. Our findings are consistent with this evidence and suggest the xanthophyll subclass significantly contribute to the beneficial effects of delaying CKD. Their study also found no significant association for both tocopherol isoforms and lycopene, for which the existing cross-sectional evidence is conflicting.

Overall, the current evidence for dietary antioxidant intervention in preventing or delaying renal dysfunction remains unclear. Inadequately powered studies, intervention duration and heterogeneity in antioxidant dosage may contribute to the lack of consistency in the findings reported from supplement interventional trials. Our findings suggest a potential future role for dietary carotenoids in early intervention - particularly those of the xanthophyll subclass. This highlights the need for an improved understanding of the exogenous antioxidant milieu in maintaining renal function to inform future interventions, particularly given the lack of cost-effective treatments for CKD. However, further studies are required to confirm our findings and elucidate the true association between renal function and exogenous antioxidants to explore the potential role of these modifiable factors in delaying or preventing disease.
